# Myasthenia Gravis presenting as a Dissociative Disorder: a case report of a differential diagnosis

**DOI:** 10.1192/j.eurpsy.2024.1698

**Published:** 2024-08-27

**Authors:** A. P. Laizāne, A. Blekte, A. Bērziņa

**Affiliations:** ^1^Riga Stradins University, Riga; ^2^Psychiatry, Hospital Ģintermuiža; ^3^Neurology, Jelgava Central Hospital, Jelgava, Latvia

## Abstract

**Introduction:**

Conversion disorder is characterised by symptoms that can impact sensory or motor function. The average incidence of conversion disorder is between 4 -12 per 100,000 per year. Conversion disorder has a wide variety of somatic and neurological differential diagnoses.

**Objectives:**

A 22-year-old woman was admitted to the hospital due to COVID-19 pneumonia. During the hospitalisation period, she developed progressive weakness, due to which she couldn’t move, eat or take care of herself. In terms of history, she is healthy, married and gave birth to her first child almost 9 months ago. Two days postpartum, the patient experienced an inability to connect with the child and provide care, as well as a decline in her mood. The husband reports episodes in which the patient had difficulties holding the child while being able to perform house chores, which required more physical strength. Two years prior to hospitalization, during stressful situations she experienced similar episodes and difficulty swallowing. While hospitalized, extensive testing was done, including an acetylcholine receptor antibody test, which was negative at first. Because of of the initially negative testing results a psychiatrist was called. On the first visit, the patient remained in a supine position and reported a lack of strength in both arms and legs, occasionally experiencing difficulty raising her head, however managed to stand up from the bed, walk independently for 5–6 meters, turn around, and, as soon as she reached the bed, descend into it. The staff reported her inability to walk earlier in the day. On the second visit, she notes that she feels tired but now can feed and take care of herself; however, some weakness persists in the proximal muscle groups. In between visits she received treatment with corticosteroids because of the COVID infection. After repeating the acetylcholine receptor antibody test, there was a positive result, and a diagnosis was established.

**Methods:**

This case report demonstrates how a somatic disorder can mimic a psychiatric one because of the overlapping symptoms and initial negative test results. While receiving symptomatic therapy with glucocorticoids due to the COVID infection, the patient’s condition improved; she began to eat and walk on her own.

**Results:**

From the psychiatric aspect, it was associated with separation from the child— a relieving of the stress factor, due to which dissociative symptoms decreased.

**Conclusions:**

Before considering a diagnosis of a dissociative disorder, a patient should be examined by other specialists according to their symptoms. A thorough neurologic and physical examination, as well as diagnostic tests, should be performed to exclude a physical pathology. Myasthenia gravis has a comorbidity with a number of psychiatric conditions and can also be very similar to a dissociative disorder, especially due to stress aggravating the symptoms of myasthenia gravis.

**Disclosure of Interest:**

None Declared

